# Supplementary material for: Enriched conditioning expands the regenerative ability of sensory neurons after spinal cord injury via neuronal intrinsic redox signaling
Source: Nat Commun. 2020 Dec 21;11:6425. doi: 10.1038/s41467-020-20179-z (PMC7752916; doi:10.1038/s41467-020-20179-z)
Supplement: Supplementary file 1 — Supplementary Information [file 41467_2020_20179_MOESM1_ESM.pdf]

SUPPLEMENTARY INFORMATION

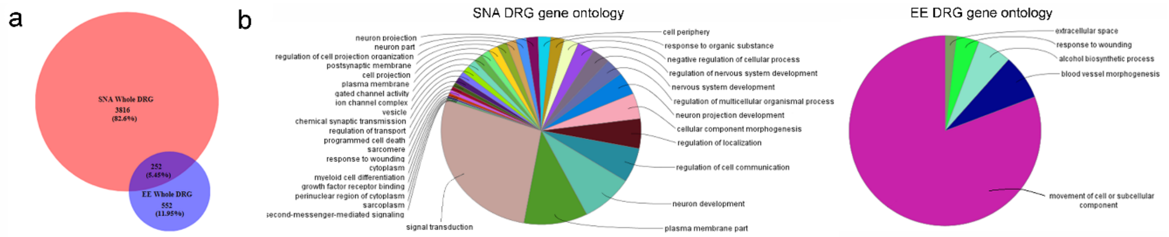

Supplementary Figure 1. a Area proportional Venn diagram showing the number of DE genes and the extent of overlap between SH SNA and EE Sham vs SH Sham P-value <0.05, n=3 independent biological animals/group). **b** Pie chart of the distribution of genes in each functional group identified in the clustering GO analysis of the DE genes upon SH SNA and EE Sham vs SH Sham run with ClueGO in Cytoscape. Functional groups are highlighted in different colours (Bonferroni P-value <0.05).

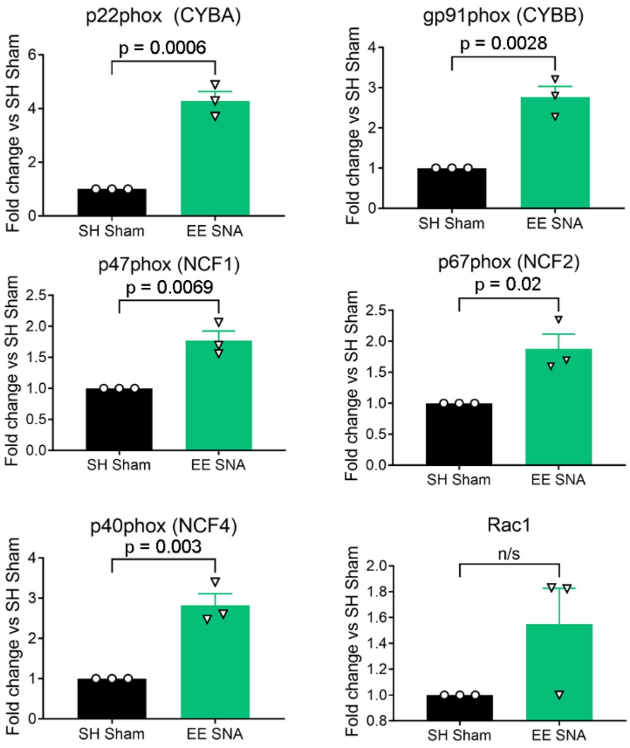

Supplementary Figure 2. Quantitative RT-PCR analysis of NOX2 complex subunits mRNA levels in DRG after SH Sham or EE+SNA (mean ± SEM, two-tailed unpaired Student's t-test, n/s = non-significant, n=3 biologically independent animals/group examined over 3 independent experiments).

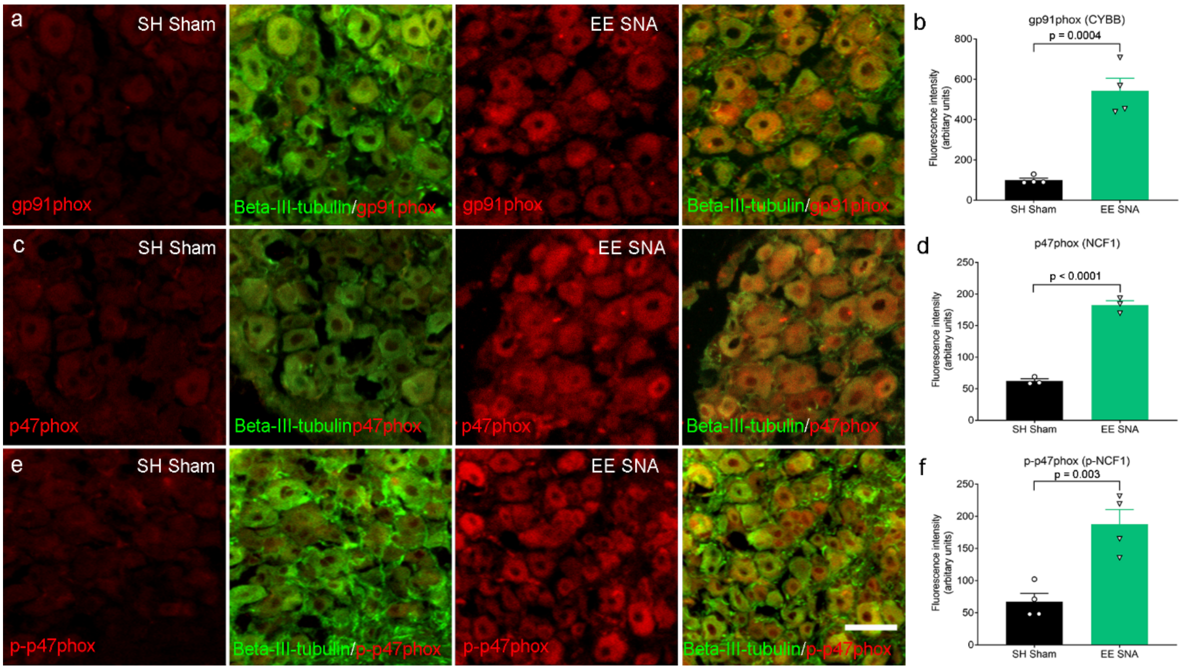

Supplementary Figure 3. Representative images of DRG immunostained for gp91phox (**a**), p47phox (**c**) or p-p47phox (**e**) (red) and Beta-III-tubulin (green) after mice were exposed to SH Sham or EE+SNA. Scale bar, 100 µm. Quantification of the fluorescence intensity of gp91phox (**b**), p47phox (**d**) or p-p47phox (**f**) in Beta-III-tubulin positive DRG neurons (mean ± SEM, two-tailed unpaired Student's t-test, n=3 or 4 biologically independent animals/group. Fluorescence intensity was measured in one series of tissue for each DRG).

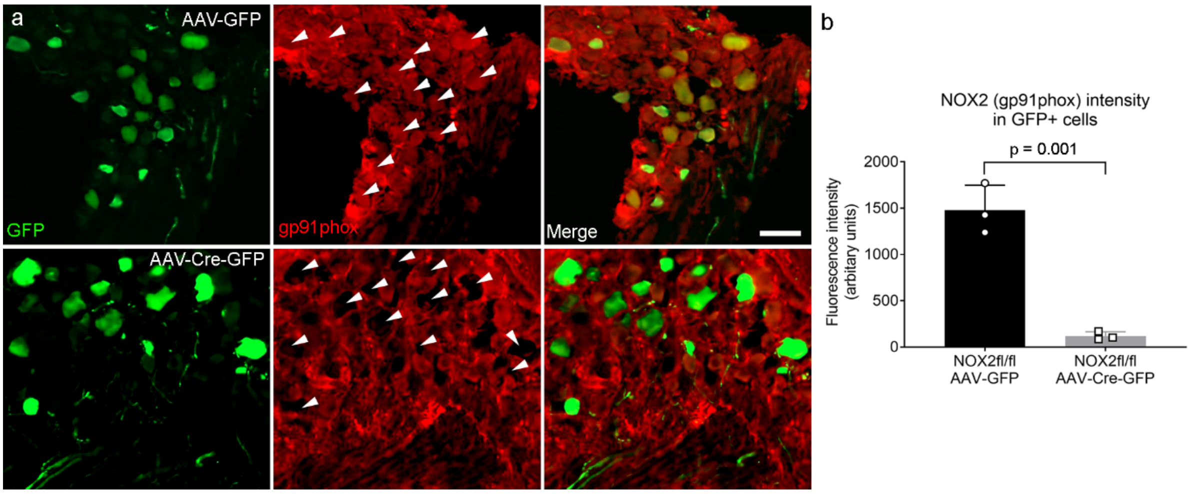

Supplementary Figure 4. a Representative images of DRG immunostained for gp91phox (red) and GFP (green) after gp91phox$^{fl/fl}$ mice were injected with AAV-GFP or AAV-Cre-GFP. Scale bar, 50 µm. **b** Quantification of the fluorescence intensity of gp91phox in GFP positive cells (mean ± SEM, two-tailed unpaired Student's t-test, n=3 biologically independent animals/group. Fluorescence intensity was measured in one series of tissue for each DRG).

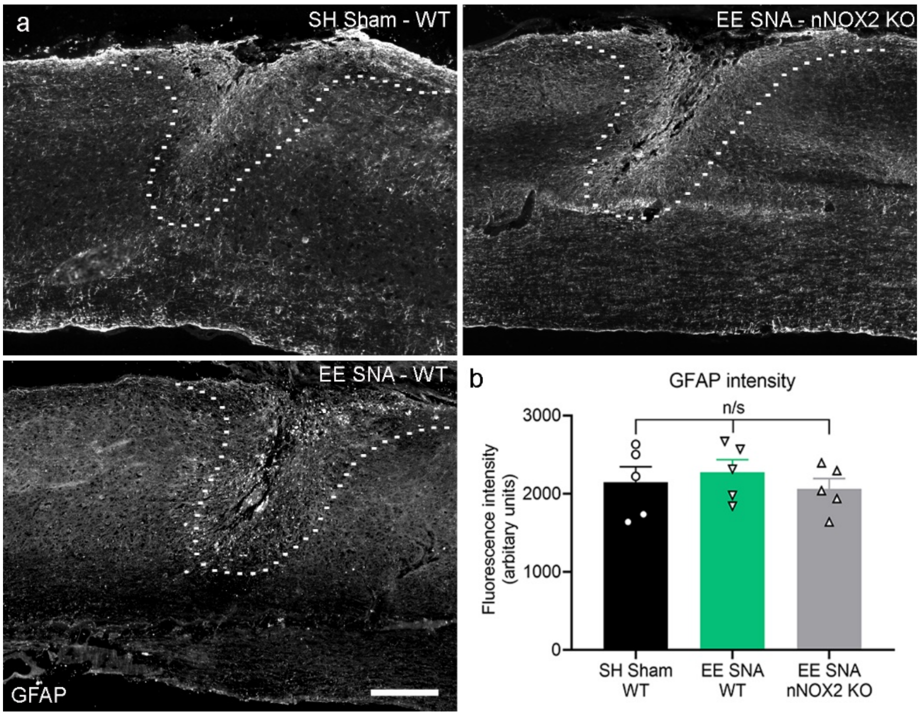

Supplementary Figure 5. a Example images of the spinal lesion site immunolabelled for GFAP to identify reactive astrocytes and the glial scar. Scale bar, 200 µm. **b** Quantification of GFAP pixel intensity around the lesion site (dotted lines) (mean ± SEM, One-way ANOVA, Tukey's post-hoc, n/s = non-significant, n = 5 biologically independent animals/group. Fluorescence intensity was measured in one series of tissue for each spinal cord).

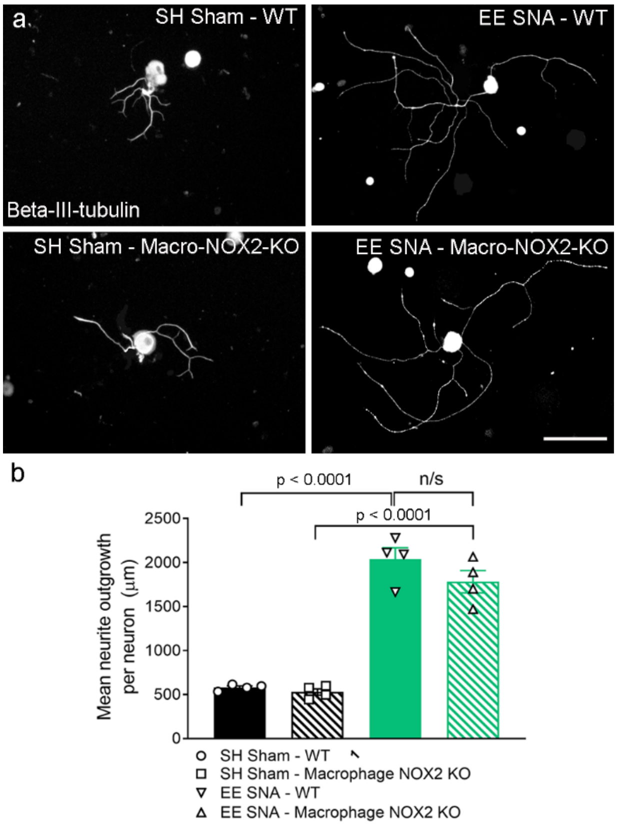

Supplementary Figure 6. a Representative images of cultured DRG from wild-type or macrophage-NOX2$^{-/-}$ mice immunostained with Beta-III-tubulin (white) after SH Sham or EE+SNA. Scale bar, 100 µm. **b** Quantification of average neurite outgrowth per neuron (mean ± SEM, One-way ANOVA, Tukey's post-hoc, n/s = non-significant, n=4 biologically independent animals/group, average of 15 cells/replicate).

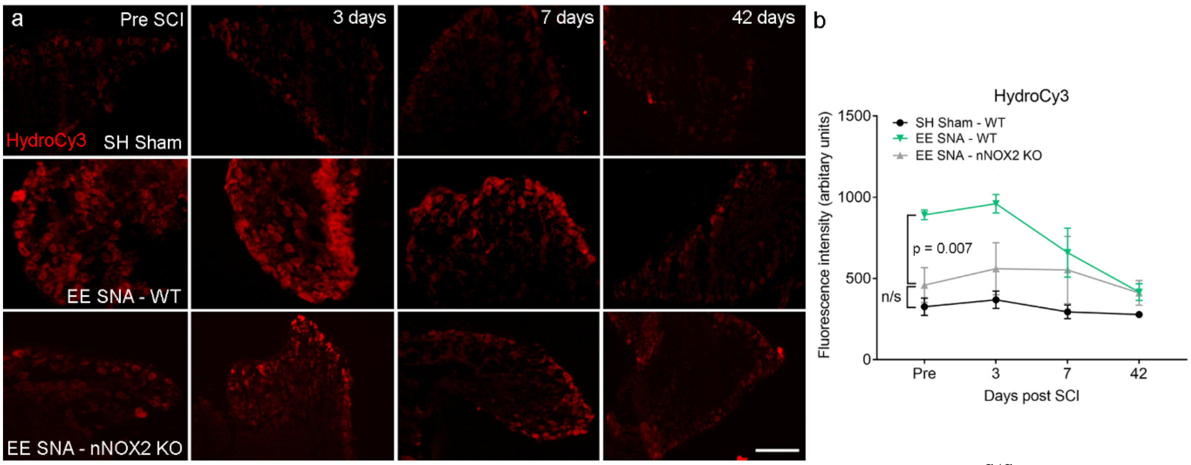

Supplementary Figure 7. a Example images of DRG from WT or NOX2$^{fl/fl}$ mice after SH Sham or EE+SNA, DRG were incubated with HydrocyanineCy3 (red) at different time points before and after a subsequent SCI. **b** Quantification of HydroCy3 levels in DRG neurons (mean ± SEM, Two-way repeated measures ANOVA, Tukey's post-hoc, n/s = non-significant, n=3 biologically independent animals/group. Fluorescence intensity was measured in one series of tissue for each DRG).

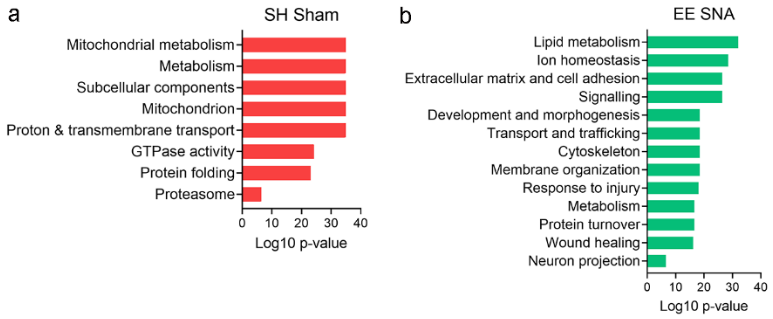

Supplementary Figure 8. a-c. Histograms of the functional groups identified in the clustering GO analysis of the differentially oxidized proteins specific for SH Sham (red, **a**) and EE+SNA (green, **b**) or common to both (grey, **c**) run with ClueGO in Cytoscape (Bonferroni P-value <0.05).

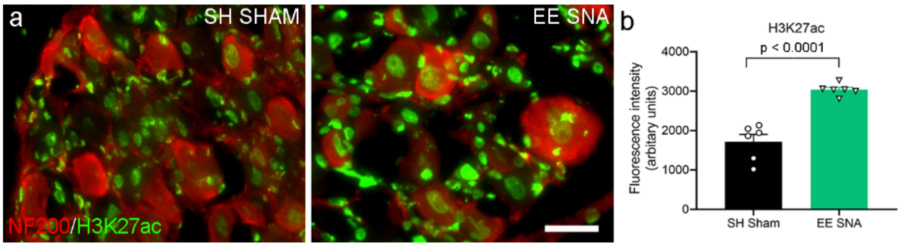

Supplementary Figure 9. a Representative images of DRG immunostained for H3K27ac (green) and NF200 (red) after mice were exposed to SH Sham or EE+SNA. Scale bar, 50 µm. **b** Quantification of the fluorescence intensity of H3K27ac in the nuclei of NF200 positive DRG neurons (mean ± SEM, two-tailed unpaired Student's t-test, n=6 biologically independent animals/group examined over 2 independent experiments. Fluorescence intensity was measured in one series of tissue for each DRG).

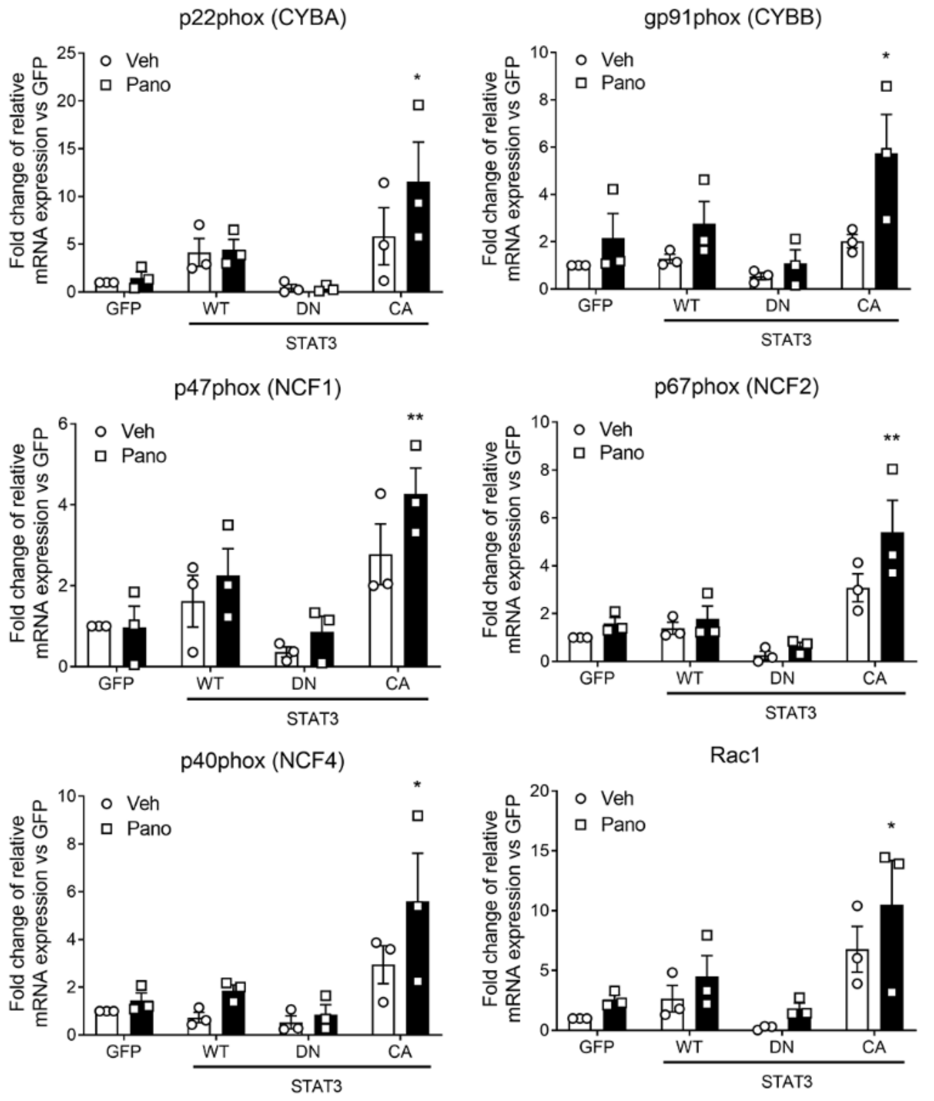

Supplementary Figure 10. Quantitative RT-PCR analysis of NOX2 complex subunits mRNA levels in HEK293T cells transfected with wild-type, constitutively active or dominant negative form of STAT3 in the presence or absence of Panobinostat (mean ± SEM, One-way ANOVA, Tukey's post-hoc * P-value <0.05; ** P-value <0.01, n=3 independent biological experiments/group).

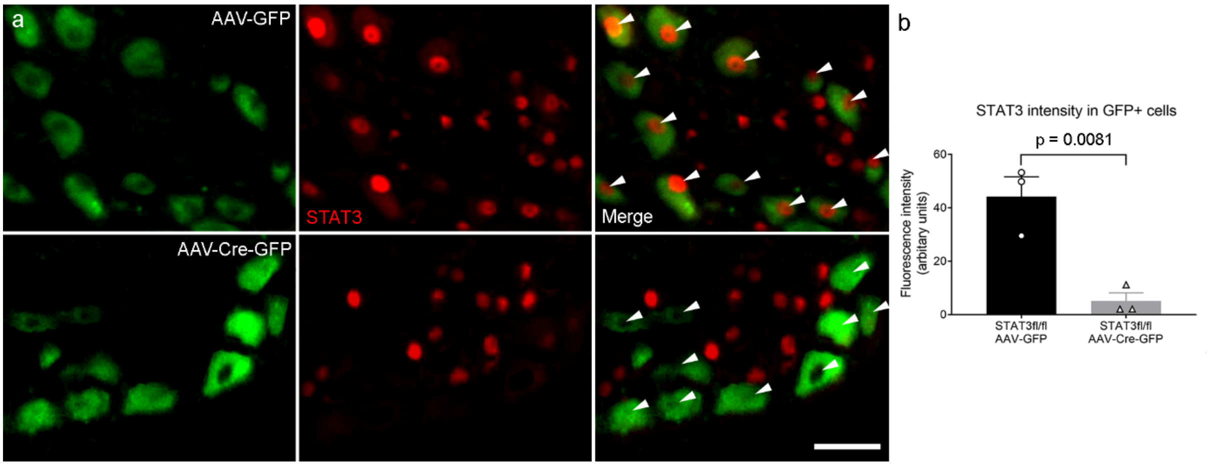

Supplementary Figure 11. a Representative images of DRG immunostained for STAT3 (red) and GFP (green) after STAT3$^{fl/fl}$ mice were injected with AAV-GFP or AAV-Cre-GFP. Arrowheads show STAT3 positive and negative labelled GFP positive cells. Scale bar, 50 µm. **b** Quantification of the fluorescence intensity of STAT3 in GFP positive cells (mean ± SEM, two-tailed unpaired Student's t-test, n=3 biologically independent animals/group. Fluorescence intensity was measured in one series of tissue for each DRG).

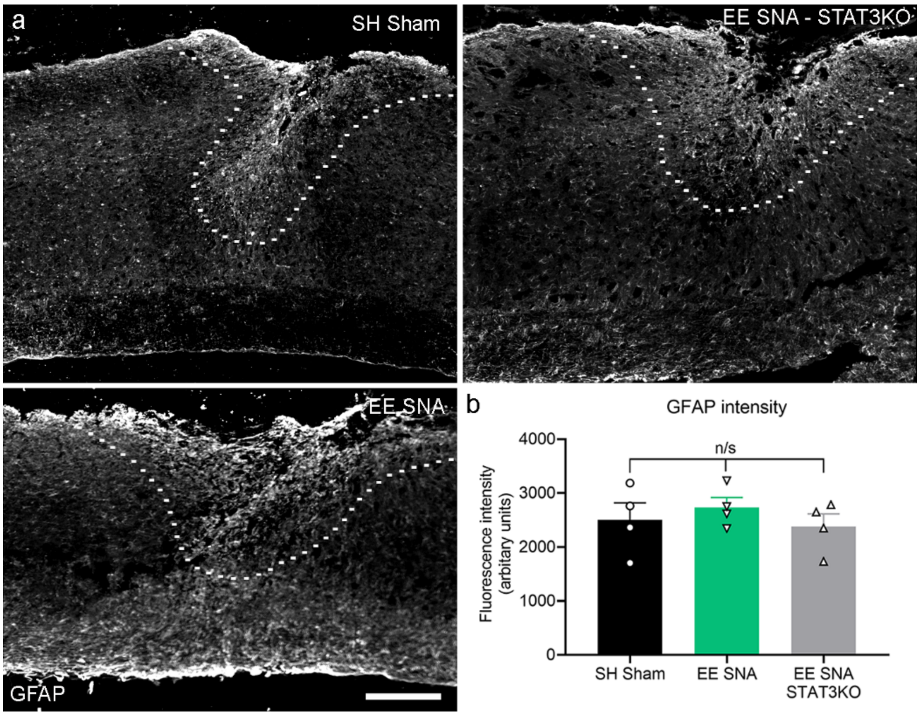

Supplementary Figure 12. a Example images of the spinal lesion site immunolabelled for GFAP to identify reactive astrocytes and the glial scar. Scale bar, 200 µm. **b** Quantification of GFAP pixel intensity around the lesion site (dotted lines) (mean ± SEM, One-way ANOVA, Tukey’s post-hoc, n/s = non-significant, n = 4 biologically independent animals/group. Fluorescence intensity was measured in one series of tissue for each spinal cord).

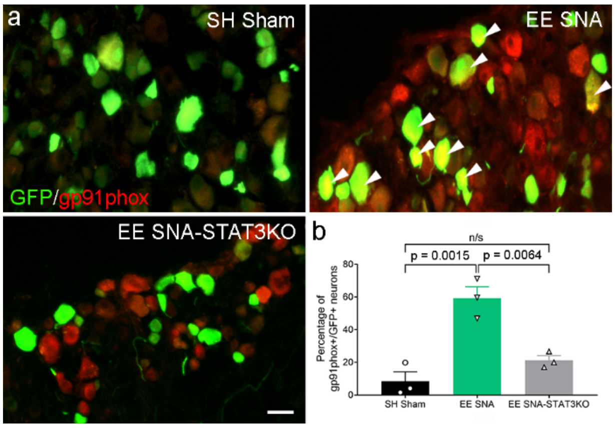

Supplementary Figure 13. **a** Representative images of transduced DRG neurons from STAT3$^{fl/fl}$ mice injected with AAV-GFP or AAV-Cre-GFP (green) and stained for gp91phox (red). Arrowheads show double labelled cells after EE+SNA. Scale bar, 50 µm. **b** Quantification of the percentage of GFP/gp91phox positive DRG neurons (mean ± SEM, One-way ANOVA, Tukey's post-hoc, n/s = non-significant, n=3 biologically independent animals/group. Percentage of GFP+ neurons was measured in one series of tissue for each DRG).

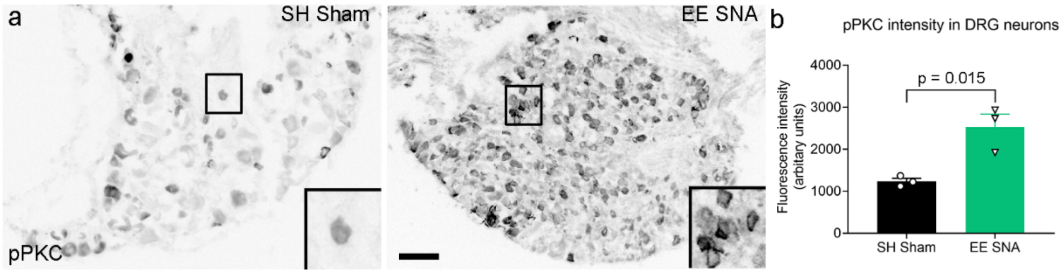

Supplementary Figure 14. **a** Representative images of DRG neurons stained for pPKC. Scale bar, 100 µm. **b** Quantification of pPKC fluorescence intensity in DRG neurons (mean ± SEM, two-tailed unpaired Student's t-test, n=3 biologically independent animals/group. Fluorescence intensity was measured in one series of tissue for each DRG).

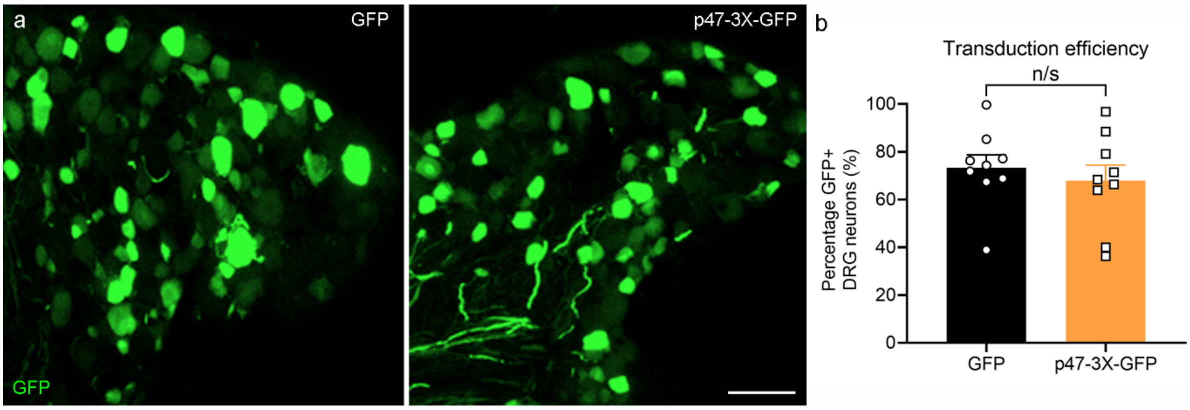

Supplementary Figure 15. **a** Representative images of DRG neurons transduced with AAV-GFP or AAV-p47-3X-GFP (green). Scale bar, 100 µm. **b** Quantification of the percentage transduction efficiency (mean ± SEM, two-tailed unpaired Student's t-test, n/s = non-significant, n=9 biologically independent animals/group examined over 2 independent experiments. Percentage of GFP+ neurons was measured in one series of tissue for each DRG).

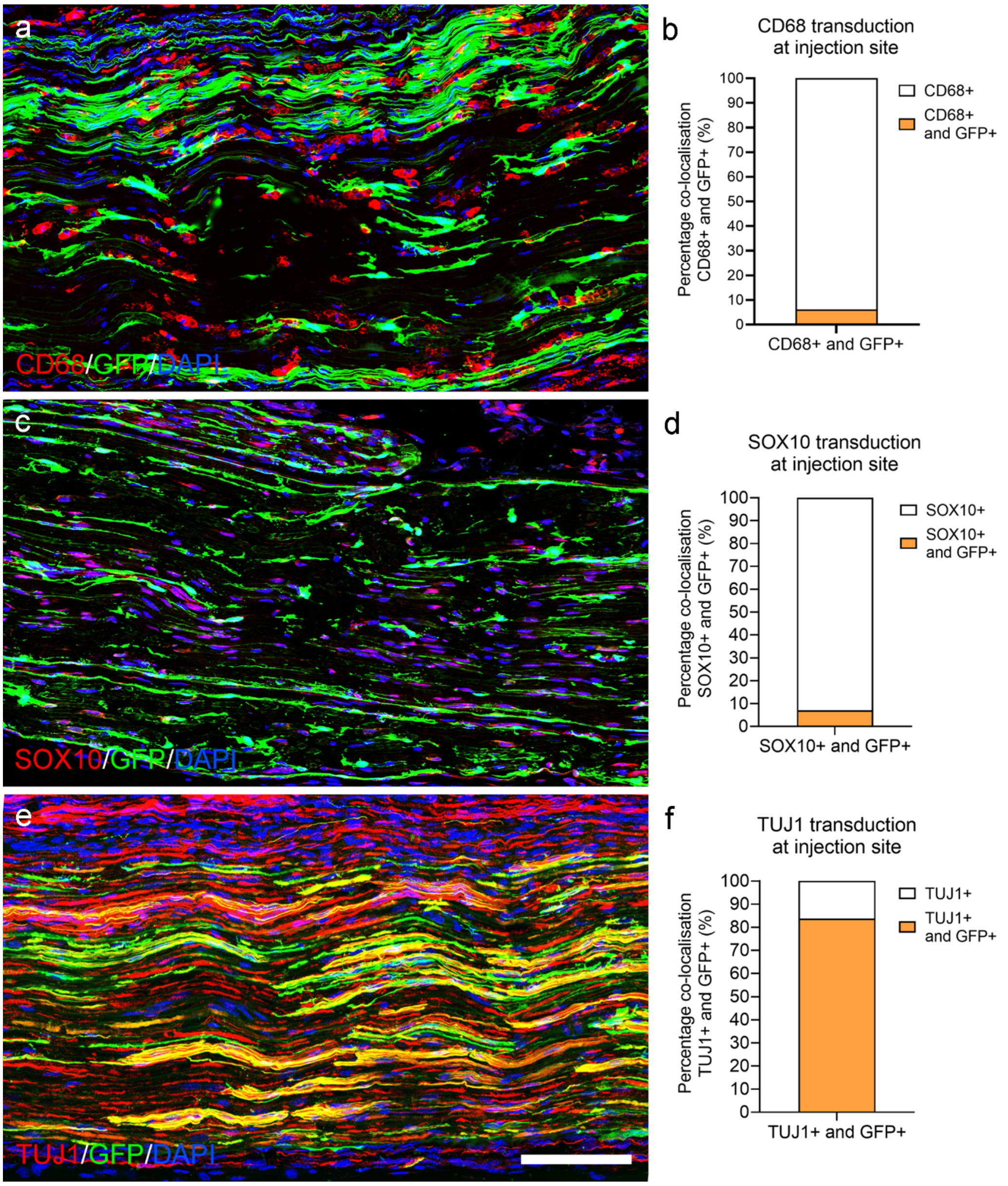

Supplementary Figure 16. a Representative confocal images of the sciatic nerve injection site four weeks after AAV8-GFP injection immunolabelled for CD68 (red) to identify macrophages, GFP (green) and DAPI (blue). **b** Quantification of the percentage of CD68+ GFP+ double positive macrophages (percentage of transduced macrophages in orange, n=3 biologically independent animals/group. Percentage of co-localisation was measured in one series of tissue for each nerve). **c** Representative confocal images of the sciatic nerve injection site four weeks after AAV8-GFP injection immunolabelled for SOX10 (red) to identify proliferating Schwann cells, GFP (green) and DAPI (blue). **d** Quantification of the percentage of SOX10+ GFP+ double positive Schwann cells (percentage of transduced Schwann cells in orange, n=3 biologically independent animals/group. Percentage of co-localisation was measured in one series of tissue for each nerve). **e** Representative confocal images of the sciatic nerve injection site four weeks after AAV8-GFP injection immunolabelled for Tuj1 (red) to identify axons, GFP (green) and DAPI (blue). Scale bar, 50 µm. **f** Quantification of the percentage of Tuj1+ GFP+ double positive axons. (percentage of transduced axons in orange, n=3 biologically independent animals/group. Percentage of co-localisation was measured in one series of tissue for each nerve).

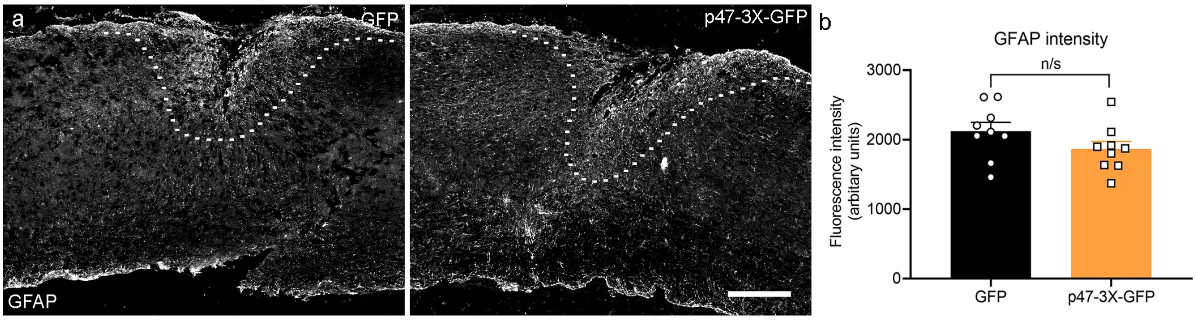

Supplementary Figure 17. a Example images of the spinal lesion site immunolabelled for GFAP to identify reactive astrocytes and the glial scar after GFP or p47-3X-GFP treatment. Scale bar, 200 µm. **b** Quantification of GFAP pixel intensity around the lesion site (mean ± SEM, two-tailed unpaired Student's t test, n/s = non-significant, n=9 biologically independent animals/group examined over 2 independent experiments. Fluorescence intensity was measured in one series of tissue for each spinal cord).

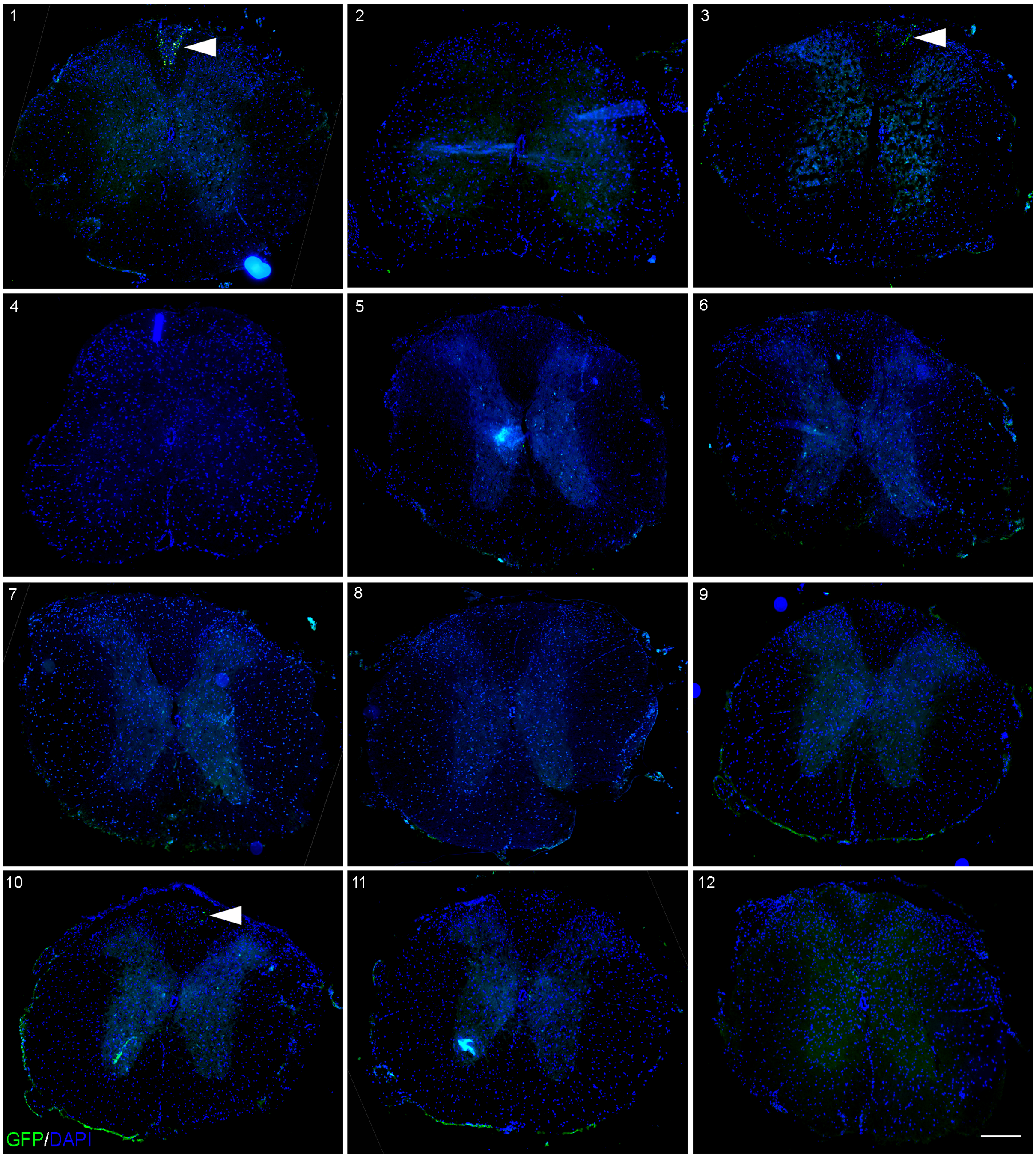

Supplementary Figure 18. Example coronal sections of T3 spinal cords (rostral to the lesion site) from AAV-p47-3X-GFP injected mice, GFP (green) and DAPI (blue). Arrowheads indicate spared axons. Scale bar, 200 μm.
