# Supplementary material for: Enriched conditioning expands the regenerative ability of sensory neurons after spinal cord injury via neuronal intrinsic redox signaling
Source: Nat Commun. 2020 Dec 21;11:6425. doi: 10.1038/s41467-020-20179-z (PMC7752916; doi:10.1038/s41467-020-20179-z)
Supplement: Supplementary file 3 — Description of Additional Supplementary Files [file 41467_2020_20179_MOESM3_ESM.pdf]

Description of Additional Supplementary Files

Supplementary Data 1: Complete RNAseq dataset of differentially regulated genes (generalized linear model (GLM) likelihood ratio test, P-value <0.05) in each indicated condition (n= 3 independent samples). For each gene, the gene Ensembl ID, the gene name, the Log2 fold change (FC), the log2 count-per-million, likelihood ratio statistics (LR), P-value is reported. No multiple comparison adjustments were made

Supplementary Data 2: GO analysis using DAVID of differentially regulated genes upon SH SNA (P-value <0.05). For each GO term, the number of enriched genes (count), the percentage in the population, the P-value (one-sided Modified Fisher Exact), the gene Ensembl ID, the number of genes in the input list (List Total), the hits in the population (Pop Hits), the total number in the population (Pop total), the Fold Enrichment, the Bonferroni, Benjamini, and FDR modified P-values for multiple comparison adjustments are shown. The Molecular Function (MF) GO categories are highlighted in yellow.

Supplementary Data 3: GO analysis using DAVID of differentially regulated genes upon EE Sham (P-value <0.05). For each GO term, the number of enriched genes (count), the percentage in the population, the P-value (one-sided Modified Fisher Exact), the gene Ensembl ID, the number of genes in the input list (List Total), the hits in the population (Pop Hits), the total number in the population (Pop total), the Fold Enrichment, the Bonferroni, Benjamini, and FDR modified P-values for multiple comparison adjustments are shown. The MF - GO categories are highlighted in yellow.

Supplementary Data 4: GO analysis using DAVID of differentially regulated genes upon EE SNA (P-value <0.05). For each GO term, the number of enriched genes (count), the percentage in the population, the P-value (one-sided Modified Fisher Exact), the gene Ensembl ID, the number of genes in the input list (List Total), the hits in the population (Pop Hits), the total number in the population (Pop total), the Fold Enrichment, the Bonferroni, Benjamini, and FDR modified P-values for multiple comparison adjustments are shown. The MF - GO categories are highlighted in yellow.

Supplementary Data 5: Statistical analysis of the network generated with the differentially regulated genes upon EE SNA (P-value <0.05) that were enriched in the GO functional clustering. Additional 4 random networks were generated as control. Shown are quantitative parameters of each network and their statistical analysis (one-sample Student t-test).

Supplementary Data 6: Statistical analysis of differentially oxidized proteins (two-sided Student t-test followed by FDR multiple comparison adjustments, FDR <0.05) in each indicated condition (N=6, independent samples). Sample correlation, principal component analysis and protein groups are also reported.

Supplementary Data 7: GO analysis using ClueGO in Cytoscape. For each GO term, GO ID, GO term, ontology source, term Pvalue, term Pvalue corrected with Bonferroni, group P-value (one-sided Fisher Exact), group P-value corrected with Bonferroni for multiple comparisons, GO levels. GO groups, % of associated genes, number of genes, cluster, genes cluster #1, genes cluster #2, % genes cluster #1, % genes cluster #2, associated genes found are reported (Bonferroni P-value <0.05). GO terms preferentially modulated by Sham, EE+SNA or common between the two are highlighted in light red, light green and light grey, respectively.
