# Supplementary material for: Enriched conditioning expands the regenerative ability of sensory neurons after spinal cord injury via neuronal intrinsic redox signaling
Source: Nat Commun. 2020 Dec 21;11:6425. doi: 10.1038/s41467-020-20179-z (PMC7752916; doi:10.1038/s41467-020-20179-z)
Supplement: Supplementary file 11 — Reporting Summary [file 41467_2020_20179_MOESM11_ESM.pdf]

nature research

Corresponding author(s): Simone Di Giovanni

Last updated by author(s): 10/22/2020

Reporting Summary

Nature Research wishes to improve the reproducibility of the work that we publish. This form provides structure for consistency and transparency in reporting. For further information on Nature Research policies, see Authors & Referees and the Editorial Policy Checklist .

Statistics

For all statistical analyses, confirm that the following items are present in the figure legend, table legend, main text, or Methods section.

n/a	Confirmed	
☐	☒	The exact sample size (*n*) for each experimental group/condition, given as a discrete number and unit of measurement
☐	☒	A statement on whether measurements were taken from distinct samples or whether the same sample was measured repeatedly
☐	☒	The statistical test(s) used AND whether they are one- or two-sided<br>*Only common tests should be described solely by name; describe more complex techniques in the Methods section.*
☒	☐	A description of all covariates tested
☐	☒	A description of any assumptions or corrections, such as tests of normality and adjustment for multiple comparisons
☐	☒	A full description of the statistical parameters including central tendency (e.g. means) or other basic estimates (e.g. regression coefficient) AND variation (e.g. standard deviation) or associated estimates of uncertainty (e.g. confidence intervals)
☐	☒	For null hypothesis testing, the test statistic (e.g. *F*, *t*, *r*) with confidence intervals, effect sizes, degrees of freedom and *P* value noted<br>*Give P values as exact values whenever suitable.*
☒	☐	For Bayesian analysis, information on the choice of priors and Markov chain Monte Carlo settings
☒	☐	For hierarchical and complex designs, identification of the appropriate level for tests and full reporting of outcomes
☒	☐	Estimates of effect sizes (e.g. Cohen's *d*, Pearson's *r*), indicating how they were calculated

Our web collection on statistics for biologists contains articles on many of the points above.

Software and code

Policy information about availability of computer code

Data collection	Nikon Eclipse TE2000 microscope and Leica SP8 confocal microscope (for image acquisition), SDS 2.4 Software v2.0 (for RT-PCR), MaxQuant Version 1.6.1.0<br>(for Mass Spectrometry)
Data analysis	RNAseq data was aligned using TopHat version 2.0.12 running Bowtie2 version 2.2.3. All datasets were aligned to the mm10 mouse reference genome. Read counts per genomic interval were calculated using HTSeq version 0.6.1.<br>Differential analysis of signal in genomic regions was performed using EdgeR version 3.8.6.<br>Gene Ontology and KEGG Pathway were generated using DAVID 6.7 (https://david-d.ncifcrf.gov/).<br>Protein-protein networks were generated with String 10.5 (https://string-db.org/) and visualized with Cytoscape 3.7.1 (http://www.cytoscape.org/).<br>Heatmap was created using GraphPad Prism 8.0<br>Venny diagrams were generated using BioVenn (C) 2007 - 2020 Tim Hulsen (http://www.biovenn.nl/).<br>ImageJ (Fiji 64bits 1.52p) Macromanager 1.4 sofware was used for image acquisition.<br>ImageJ (Fiji 64bits 1.52p) NeuronJ plug-in software was used to calculate average neurite outgrowth.<br>ImageJ (Fiji 64bits 1.52p) was used to quantify the sprouting and regrowing axon fibers.<br>ImageJ (Fiji 64bits 1.52p) was used for the fluorescence intensity and Western Blot band intensity measurement.<br>All charts were generated using GraphPad Prism 8.0.

For manuscripts utilizing custom algorithms or software that are central to the research but not yet described in published literature, software must be made available to editors/reviewers. We strongly encourage code deposition in a community repository (e.g. GitHub). See the Nature Research guidelines for submitting code & software for further information.

Data

Policy information about availability of data

All manuscripts must include a data availability statement. This statement should provide the following information, where applicable:

- Accession codes, unique identifiers, or web links for publicly available datasets
- A list of figures that have associated raw data
- A description of any restrictions on data availability

RNA sequencing data have been deposited to GEO (number :GSE138769, token: udaxemosxbejluh)
Oxiproteomics followed by Mass spectrometry data have been deposited to the ProteomeXchange Consortium via the PRIDE83 partner repository with the datasets identifiers as follows:
PROJECT NAME: Quantification of oxidized proteins in wild type and Nox2 deficient mouse dorsal root ganglia
PROJECT ACCESSION: PXD018085
Reviewer account details:
Username: reviewer70885@ebi.ac.uk.
Password: ToUnC7um

Field-specific reporting

Please select the one below that is the best fit for your research. If you are not sure, read the appropriate sections before making your selection.

☒ Life sciences ☐ Behavioural & social sciences ☐ Ecological, evolutionary & environmental sciences

For a reference copy of the document with all sections, see nature.com/documents/nr-reporting-summary-flat.pdf

Life sciences study design

All studies must disclose on these points even when the disclosure is negative.

Sample size	Sample size was either calculated using AEEC power calculator (http://www.lasec.cuhk.edu.hk/sample-size-calculation.html) to estimate the number of replicates required, for a difference of 1.5 and 80% to 90% power assuming a 5% significance level, or based upon similar previously established experimental designs.
Data exclusions	no data was excluded
Replication	To verify reproducibility, 3-9 biological replicates were used for each experiment. The exact N is explicitly given in the legend of each experiment. All attempts of replication were successful.
Randomization	The biological groups were homogeneous in terms of age (6-10 weeks) and mice. Male and female animals were allocated equally for all the experiments, unless otherwise stated.
Blinding	Dorsal column regeneration, behavioural test, DRG neurite outgrowth, western immunoblotting, qPCR analysis and fluorescence intensity measurements were performed in blind to group allocation and analysis

Behavioural & social sciences study design

All studies must disclose on these points even when the disclosure is negative.

Study description	*Briefly describe the study type including whether data are quantitative, qualitative, or mixed-methods (e.g. qualitative cross-sectional, quantitative experimental, mixed-methods case study).*
Research sample	*State the research sample (e.g. Harvard university undergraduates, villagers in rural India) and provide relevant demographic information (e.g. age, sex) and indicate whether the sample is representative. Provide a rationale for the study sample chosen. For studies involving existing datasets, please describe the dataset and source.*
Sampling strategy	*Describe the sampling procedure (e.g. random, snowball, stratified, convenience). Describe the statistical methods that were used to predetermine sample size OR if no sample-size calculation was performed, describe how sample sizes were chosen and provide a rationale for why these sample sizes are sufficient. For qualitative data, please indicate whether data saturation was considered, and what criteria were used to decide that no further sampling was needed.*
Data collection	*Provide details about the data collection procedure, including the instruments or devices used to record the data (e.g. pen and paper, computer, eye tracker, video or audio equipment) whether anyone was present besides the participant(s) and the researcher, and whether the researcher was blind to experimental condition and/or the study hypothesis during data collection.*
Timing	*Indicate the start and stop dates of data collection. If there is a gap between collection periods, state the dates for each sample cohort.*

Data exclusions	*If no data were excluded from the analyses, state so OR if data were excluded, provide the exact number of exclusions and the rationale behind them, indicating whether exclusion criteria were pre-established.*
Non-participation	*State how many participants dropped out/declined participation and the reason(s) given OR provide response rate OR state that no participants dropped out/declined participation.*
Randomization	*If participants were not allocated into experimental groups, state so OR describe how participants were allocated to groups, and if allocation was not random, describe how covariates were controlled.*

Ecological, evolutionary & environmental sciences study design

All studies must disclose on these points even when the disclosure is negative.

Study description	*Briefly describe the study. For quantitative data include treatment factors and interactions, design structure (e.g. factorial, nested, hierarchical), nature and number of experimental units and replicates.*
Research sample	*Describe the research sample (e.g. a group of tagged Passer domesticus, all Stenocereus thurberi within Organ Pipe Cactus National Monument), and provide a rationale for the sample choice. When relevant, describe the organism taxa, source, sex, age range and any manipulations. State what population the sample is meant to represent when applicable. For studies involving existing datasets, describe the data and its source.*
Sampling strategy	*Note the sampling procedure. Describe the statistical methods that were used to predetermine sample size OR if no sample-size calculation was performed, describe how sample sizes were chosen and provide a rationale for why these sample sizes are sufficient.*
Data collection	*Describe the data collection procedure, including who recorded the data and how.*
Timing and spatial scale	*Indicate the start and stop dates of data collection, noting the frequency and periodicity of sampling and providing a rationale for these choices. If there is a gap between collection periods, state the dates for each sample cohort. Specify the spatial scale from which the data are taken*
Data exclusions	*If no data were excluded from the analyses, state so OR if data were excluded, describe the exclusions and the rationale behind them, indicating whether exclusion criteria were pre-established.*
Reproducibility	*Describe the measures taken to verify the reproducibility of experimental findings. For each experiment, note whether any attempts to repeat the experiment failed OR state that all attempts to repeat the experiment were successful.*
Randomization	*Describe how samples/organisms/participants were allocated into groups. If allocation was not random, describe how covariates were controlled. If this is not relevant to your study, explain why.*
Blinding	*Describe the extent of blinding used during data acquisition and analysis. If blinding was not possible, describe why OR explain why blinding was not relevant to your study.*

Did the study involve field work? ☐ Yes ☐ No

Field work, collection and transport

Field conditions	*Describe the study conditions for field work, providing relevant parameters (e.g. temperature, rainfall).*
Location	*State the location of the sampling or experiment, providing relevant parameters (e.g. latitude and longitude, elevation, water depth).*
Access and import/export	*Describe the efforts you have made to access habitats and to collect and import/export your samples in a responsible manner and in compliance with local, national and international laws, noting any permits that were obtained (give the name of the issuing authority, the date of issue, and any identifying information).*
Disturbance	*Describe any disturbance caused by the study and how it was minimized.*

Reporting for specific materials, systems and methods

We require information from authors about some types of materials, experimental systems and methods used in many studies. Here, indicate whether each material, system or method listed is relevant to your study. If you are not sure if a list item applies to your research, read the appropriate section before selecting a response.

Materials & experimental systems

n/a	Involved in the study
	☒ Antibodies
	☒ Eukaryotic cell lines
☒	☐ Palaeontology
	☒ Animals and other organisms
☒	☐ Human research participants
☒	☐ Clinical data

Methods

n/a	Involved in the study
☒	☐ ChIP-seq
☒	☐ Flow cytometry
☒	☐ MRI-based neuroimaging

Antibodies

Antibodies used

Anti-gp91phox (Santa Cruz Biotech, Sc-74514)
Anti-gp91phox (BD Bioscince, 611415, lot 5009657)
Anti-p47phox (Santa Cruz Biotech, Sc-14015)
Anti-p-p47phox (S345) (Sigma, sab4504721)
Anti-p67phox (Abcam, ab175293)
Anti-p22phox (Abcam, ab191512)
Anti-STAT3 (Cell Signalling Tech, 9139S lot 10)
Anti-p-STAT3 (Cell Signalling Tech, 9145)
Anti-p-PKC (Cell Signalling Tech, 9371)
Anti-CTB (List Biological, 703)
Anti-vGlut1 (Synaptic System, 135302)
Anti-vGAT1 (Synaptic System, 131011)
Anti-ChAT (Millipore, AB144P)
Anti-H3K27ac (Abcam, ab4729)
Anti-GAPDH (Cell Signaling Technology, #2118S, lot 10)
Anti-βIII Tubulin or TUJ1 (Promega, G712A, lot 0000317682)
Anti-Neurofilment 200 (Sigma, N5389, lot 118M4796V)
Anti-GFAP (Millipore, AB5804)
Anti-SOX10 (Abcam, ab264405)
Alexa Fluor 488 Goat anti Rabbit (ThermoFisher A32731)
Alexa Fluor 488 Goat anti Mouse (ThermoFisher A32723)
Alexa Fluor 488 Donkey anti Rabbit (ThermoFisher A32754)
Alexa Fluor 488 Donkey anti Mouse (ThermoFisher A32744)
HRP-conjugated anti Rabbit (GE Healthcare NA934V)
HRP-conjugated anti Mouse (GE Healthcare NA931V)

Validation

Anti-gp91phox (Santa Cruz Biotech, Sc-74514) was validated for western blot in mouse tissue (PMID: # 28532855) and (PMID: 29520084), we further validated this antibody in Figure 2.
Anti-gp91phox (BD Bioscince, 611415, lot 5009657) was validated for immunostaining in mouse tissue (PMID: 29520084), we further validated this antibody in Supplementary Figure 3.
Anti-p47phox (Santa Cruz Biotech, Sc-14015) was validated for western blot and immunostaining in mouse tissue (PMID: 31391506) and (PMID: 29520084), we further validated this antibody in Figure 2 and Supplementary Figure 3.
Anti-p-p47phox (S345) (Sigma, sab4504721) was validated for western blot and immunostaining in mouse tissue (PMID 28183701) and (PMID: 29520084), we further validated this antibody in Figure 2 and Supplementary Figure 3.
Anti-p67phox (Abcam, ab175293) was validated for western blot in mouse tissue (PMID: 29520084), we further validated this antibody in Figure 2.
Anti-p22phox (Abcam, ab191512) was validated for western blot in mouse tissue (PMID: 30151070), we further validated this antibody in Figure 2.
Anti-STAT3 (Cell Signalling Tech, 9139S lot 10) was validated for immunostaining in mouse tissue (PMID: 9108058), we further validated this antibody in Figure 5 and Supplementary Figure 11.
Anti-p-STAT3 (Cell Signalling Tech, 9145) was validated for immunostaining in mouse tissue (PMID: 9108058), we further validated this antibody in Figure 5 and 6.
Anti-p-PKC (Cell Signalling Tech, 9371) was validated for western blot in human tissue (PMID: 31308409) and in mouse tissue (PMID: 31346193), we further validated this antibody in Supplementary Figure 14.
Anti-CTB (List Biological, 703) was validated for immunostaining in mouse tissue (PMID: 30971452), we further validated this antibody in Figure 1, 3 and 6.
Anti-vGlut1 (Synaptic System, 135302) was validated for western blot and immunostaining in mouse tissue (PMID: 3027945) and (PMID: 28864826), we further validated this antibody in Figure 7.
Anti-vGAT1 (Synaptic System, 131011) was validated for western blot and immunostaining in mouse tissue (PMID: 32051550) and (PMID: 31782729), we further validated this antibody in Figure 7.
Anti-ChAT (Millipore, AB144P) was validated for immunostaining in mouse tissue (PMID: 30971452), we further validated this antibody in Figure 7.
Anti-GAPDH (Cell Signaling Technology, #2118S) was validated for western blot in mouse tissue (http://europepmc.org/backend/

ptpmcrender.fcgi?accid=PMC6534559&blobtype=pdf and http://europepmc.org/backend/ptpmcrender.fcgi?accid=PMC6525201&blobtype=pdf) and we further validated this antibody in Figure 2.
Anti-βIII Tubulin or TUJ1 (Promega, G712A) was validated for western blot in rat DRG neurons (https://www.sciencedirect.com/science/article/pii/S2452073X18300163) and we further validated this antibody in Figure 1,3 and 6.
Anti-Neurofilment 200 (Sigma, N5389) was validated for IF in mouse ventral diaphragm (https://elifesciences.org/articles/14371) and we further validated this antibody in Supplementary Figure 9.
Anti-GFAP (Millipore, AB5804) was validated for IF in mouse spinal cord (https://stm.sciencemag.org/content/11/487/eaaw2064/tab-pdf) and we further validated this antibody in Figure 1 and Supplementary Figure 5, 12 and 17.
Anti-SOX10 (Abcam, ab264405) was validated for IF in the sciatic nerve in Supplementary Figure 16.

Eukaryotic cell lines

Policy information about cell lines

Cell line source(s)	HEK293 cell lines was purchased for Sigma-Aldrich.
Authentication	Morphological evaluation of the cells was used as authentication method.
Mycoplasma contamination	No mycoplasma contamination
Commonly misidentified lines (See ICLAC register)	No commonly misidentified cell lines was used

Palaeontology

Specimen provenance	*Provide provenance information for specimens and describe permits that were obtained for the work (including the name of the issuing authority, the date of issue, and any identifying information).*
Specimen deposition	*Indicate where the specimens have been deposited to permit free access by other researchers.*
Dating methods	*If new dates are provided, describe how they were obtained (e.g. collection, storage, sample pretreatment and measurement), where they were obtained (i.e. lab name), the calibration program and the protocol for quality assurance OR state that no new dates are provided.*

☐ Tick this box to confirm that the raw and calibrated dates are available in the paper or in Supplementary Information.

Animals and other organisms

Policy information about studies involving animals; ARRIVE guidelines recommended for reporting animal research

Laboratory animals	All animal procedures were performed in accordance to Home Office under the Animals (Scientific Procedures) Act 1986, with Local Ethical Review by the Imperial College London Animal Welfare and Ethical Review Body Standing Committee (AWERB). Three mouse germ-lines were used for this study: C57BL6/J (Charles River Laboratories), Stat3flox (Stock No: 000664) and Nox2flox (Stock No: 031777) mice (purchased from The Jackson Laboratory). Mice ranging from 6-10 weeks age were used for all the experiments. Males and females were allocated equally for all the experiments, unless otherwise stated.
Wild animals	The study did not involve wild animals.
Field-collected samples	The study did not involve samples collected from the field.
Ethics oversight	Animal work was carried out in accordance to Home Office under the Animals (Scientific Procedures) Act 1986, with Local Ethical Review by the Imperial College London Animal Welfare and Ethical Review Body Standing Committee (AWERB).

Note that full information on the approval of the study protocol must also be provided in the manuscript.

Human research participants

Policy information about studies involving human research participants

Population characteristics	*Describe the covariate-relevant population characteristics of the human research participants (e.g. age, gender, genotypic information, past and current diagnosis and treatment categories). If you filled out the behavioural & social sciences study design questions and have nothing to add here, write "See above."*
Recruitment	*Describe how participants were recruited. Outline any potential self-selection bias or other biases that may be present and how these are likely to impact results.*
Ethics oversight	*Identify the organization(s) that approved the study protocol.*

Note that full information on the approval of the study protocol must also be provided in the manuscript.

Clinical data

Policy information about clinical studies

All manuscripts should comply with the ICMJEguidelines for publication of clinical research and a completedCONSORT checklist must be included with all submissions.

Clinical trial registration	*Provide the trial registration number from ClinicalTrials.gov or an equivalent agency.*
Study protocol	*Note where the full trial protocol can be accessed OR if not available, explain why.*
Data collection	*Describe the settings and locales of data collection, noting the time periods of recruitment and data collection.*
Outcomes	*Describe how you pre-defined primary and secondary outcome measures and how you assessed these measures.*

ChIP-seq

Data deposition

☐ Confirm that both raw and final processed data have been deposited in a public database such as GEO.

☐ Confirm that you have deposited or provided access to graph files (e.g. BED files) for the called peaks.

Data access links<br>*May remain private before publication.*	*For "Initial submission" or "Revised version" documents, provide reviewer access links. For your "Final submission" document, provide a link to the deposited data.*
Files in database submission	*Provide a list of all files available in the database submission.*
Genome browser session<br>(e.g. UCSC)	*Provide a link to an anonymized genome browser session for "Initial submission" and "Revised version" documents only, to enable peer review. Write "no longer applicable" for "Final submission" documents.*

Methodology

Replicates	*Describe the experimental replicates, specifying number, type and replicate agreement.*
Sequencing depth	*Describe the sequencing depth for each experiment, providing the total number of reads, uniquely mapped reads, length of reads and whether they were paired- or single-end.*
Antibodies	*Describe the antibodies used for the ChIP-seq experiments; as applicable, provide supplier name, catalog number, clone name, and lot number.*
Peak calling parameters	*Specify the command line program and parameters used for read mapping and peak calling, including the ChIP, control and index files used.*
Data quality	*Describe the methods used to ensure data quality in full detail, including how many peaks are at FDR 5% and above 5-fold enrichment.*
Software	*Describe the software used to collect and analyze the ChIP-seq data. For custom code that has been deposited into a community repository, provide accession details.*

Flow Cytometry

Plots

Confirm that:

☐ The axis labels state the marker and fluorochrome used (e.g. CD4-FITC).

☐ The axis scales are clearly visible. Include numbers along axes only for bottom left plot of group (a 'group' is an analysis of identical markers).

☐ All plots are contour plots with outliers or pseudocolor plots.

☐ A numerical value for number of cells or percentage (with statistics) is provided.

Methodology

Sample preparation	*Describe the sample preparation, detailing the biological source of the cells and any tissue processing steps used.*
Instrument	*Identify the instrument used for data collection, specifying make and model number.*
Software	*Describe the software used to collect and analyze the flow cytometry data. For custom code that has been deposited into a community repository, provide accession details.*

Cell population abundance	*Describe the abundance of the relevant cell populations within post-sort fractions, providing details on the purity of the samples and how it was determined.*
Gating strategy	*Describe the gating strategy used for all relevant experiments, specifying the preliminary FSC/SSC gates of the starting cell population, indicating where boundaries between "positive" and "negative" staining cell populations are defined.*

☐ Tick this box to confirm that a figure exemplifying the gating strategy is provided in the Supplementary Information.

Magnetic resonance imaging

Experimental design

Design type	*Indicate task or resting state; event-related or block design.*
Design specifications	*Specify the number of blocks, trials or experimental units per session and/or subject, and specify the length of each trial or block (if trials are blocked) and interval between trials.*
Behavioral performance measures	*State number and/or type of variables recorded (e.g. correct button press, response time) and what statistics were used to establish that the subjects were performing the task as expected (e.g. mean, range, and/or standard deviation across subjects).*

Acquisition

Imaging type(s)	*Specify: functional, structural, diffusion, perfusion.*
Field strength	*Specify in Tesla*
Sequence & imaging parameters	*Specify the pulse sequence type (gradient echo, spin echo, etc.), imaging type (EPI, spiral, etc.), field of view, matrix size, slice thickness, orientation and TE/TR/flip angle.*
Area of acquisition	*State whether a whole brain scan was used OR define the area of acquisition, describing how the region was determined.*

Diffusion MRI ☐ Used ☐ Not used

Preprocessing

Preprocessing software	*Provide detail on software version and revision number and on specific parameters (model/functions, brain extraction, segmentation, smoothing kernel size, etc.).*
Normalization	*If data were normalized/standardized, describe the approach(es): specify linear or non-linear and define image types used for transformation OR indicate that data were not normalized and explain rationale for lack of normalization.*
Normalization template	*Describe the template used for normalization/transformation, specifying subject space or group standardized space (e.g. original Talairach, MNI305, ICBM152) OR indicate that the data were not normalized.*
Noise and artifact removal	*Describe your procedure(s) for artifact and structured noise removal, specifying motion parameters, tissue signals and physiological signals (heart rate, respiration).*
Volume censoring	*Define your software and/or method and criteria for volume censoring, and state the extent of such censoring.*

Statistical modeling & inference

Model type and settings	*Specify type (mass univariate, multivariate, RSA, predictive, etc.) and describe essential details of the model at the first and second levels (e.g. fixed, random or mixed effects; drift or auto-correlation).*
Effect(s) tested	*Define precise effect in terms of the task or stimulus conditions instead of psychological concepts and indicate whether ANOVA or factorial designs were used.*

Specify type of analysis: ☐ Whole brain ☐ ROI-based ☐ Both

Statistic type for inference (See Eklund et al. 2016)	*Specify voxel-wise or cluster-wise and report all relevant parameters for cluster-wise methods.*
Correction	*Describe the type of correction and how it is obtained for multiple comparisons (e.g. FWE, FDR, permutation or Monte Carlo).*

Models & analysis

n/a	Involved in the study
☐	☐ Functional and/or effective connectivity
☐	☐ Graph analysis
☐	☐ Multivariate modeling or predictive analysis

Functional and/or effective connectivity	*Report the measures of dependence used and the model details (e.g. Pearson correlation, partial correlation, mutual information).*
Graph analysis	*Report the dependent variable and connectivity measure, specifying weighted graph or binarized graph, subject- or group-level, and the global and/or node summaries used (e.g. clustering coefficient, efficiency, etc.).*
Multivariate modeling and predictive analysis	*Specify independent variables, features extraction and dimension reduction, model, training and evaluation metrics.*
